# Supplementary material for: Single AAV-mediated mutation replacement genome editing in limited number of photoreceptors restores vision in mice
Source: Nat Commun. 2020 Jan 24;11:482. doi: 10.1038/s41467-019-14181-3 (PMC6981188; doi:10.1038/s41467-019-14181-3)
Supplement: Supplementary file 1 — Supplementary Information [file 41467_2019_14181_MOESM1_ESM.pdf]

Supplementary Information

Single AAV-mediated mutation replacement genome editing in limited number of photoreceptors restores vision in mice

Nishiguchi KM *et al*.

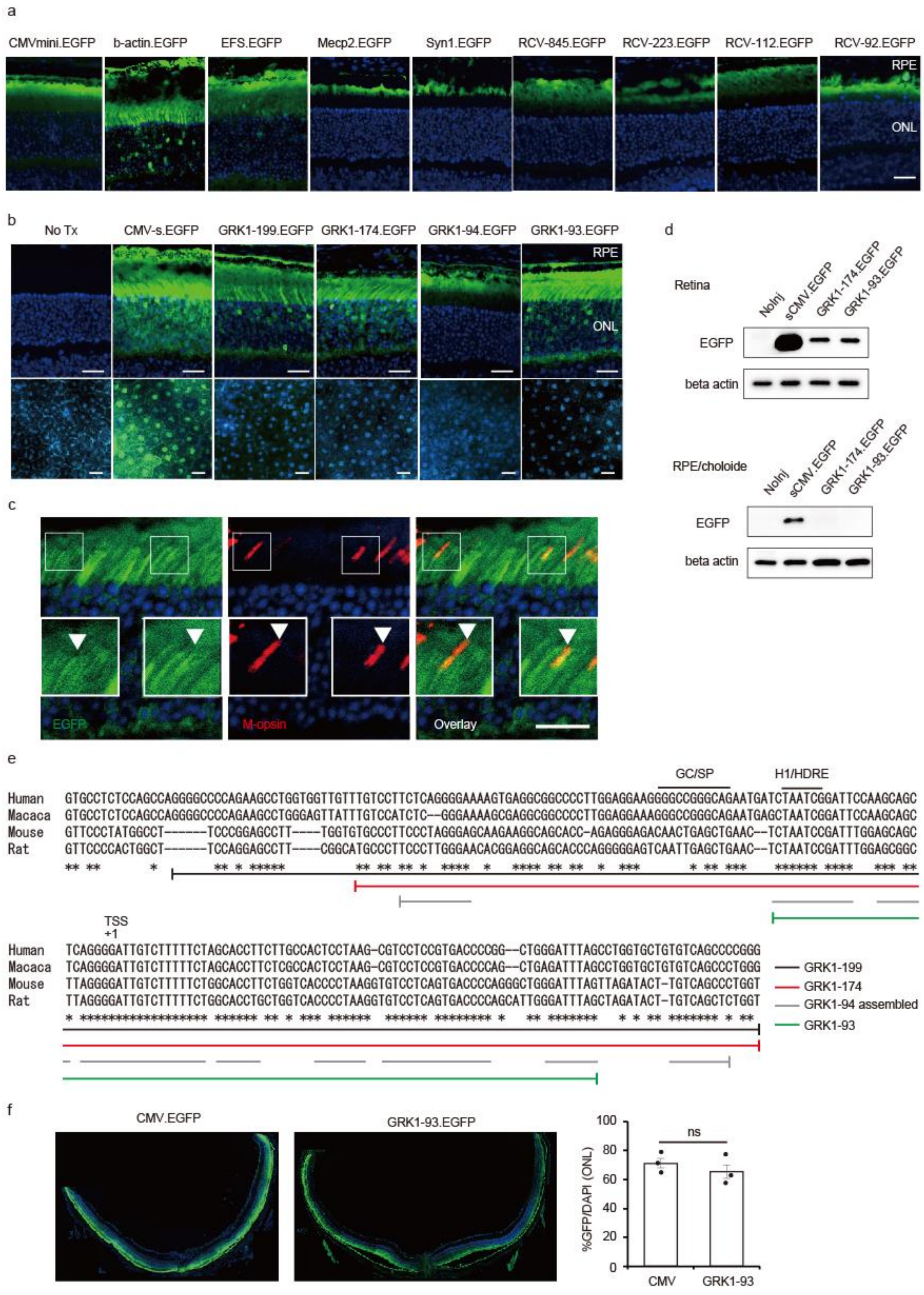
a
CMVmini.EGFP
b-actin.EGFP
EFS.EGFP
Mecp2.EGFP
Syn1.EGFP
RCV-845.EGFP
RCV-223.EGFP
RCV-112.EGFP
RCV-92.EGFP
RPE
ONL
b
No Tx
CMV-s.EGFP
GRK1-199.EGFP
GRK1-174.EGFP
GRK1-94.EGFP
GRK1-93.EGFP
RPE
ONL
c
EGFP
M-opsin
Overlay
d
Retina
NoInj
sCMV.EGFP
GRK1-174.EGFP
GRK1-93.EGFP
EGFP
beta actin
RPE/choloide
NoInj
sCMV.EGFP
GRK1-174.EGFP
GRK1-93.EGFP
EGFP
beta actin
e
GC/SP
H1/HDRE
Human
Macaca
Mouse
Rat
TSS
+1
Human
Macaca
Mouse
Rat
GRK1-199
GRK1-174
GRK1-94 assembled
GRK1-93
f
CMV.EGFP
GRK1-93.EGFP
%GFP/DAPI (ONL)
100
80
60
40
20
0
ns
CMV
GRK1-93

Supplementary Fig. 1| Selection of minimal neural retina-specific promoters

a. *In vivo* EGFP reporter analysis of retinal sections, performed three weeks after AAV injection with various published and modified promoters (N = 2 each). The ONL coincides with the photoreceptor cell bodies. The details of the promoters used are shown in supplementary Table 1. The sections were stained only with DAPI.

b. *In vivo* EGFP reporter analysis of *GRK1* promotor deletion mutants in retinal sections (upper panels) and retinal pigment epithelium flat mounts (lower panels). The sections and flat mounts were stained only with DAPI.

c. Immunohistochemistry of cone photoreceptors. *GRK1-93*-driven EGFP and cone-specific M-opsin were co-localized.

d. Western blot analysis of reporter EGFP. Note that *GRK1-93*-driven EGFP was not detected in the RPE.

e. Sequence of the *GRK1* promoter mutants tested in the experiment.

f. Comparison of transduction efficacy, based on reporter EGFP expression (green) driven by a CMV promoter or *GRK1-93* promoter, in histological sections of the eye. The proportion of photoreceptors transduced with AAV (EGFP-positive cells/DAPI positive cells in ONL, N = 3 each) is shown.

ONL, outer nuclear layer; EGFP, enhanced green fluorescent protein; NoInj, not injected. GC/SP, GC-rich regions presumably interacting with Sp protein; H1/HDRE, potential homeodomain (Crx) binding site. Source data are provided as a Source Data file.

a

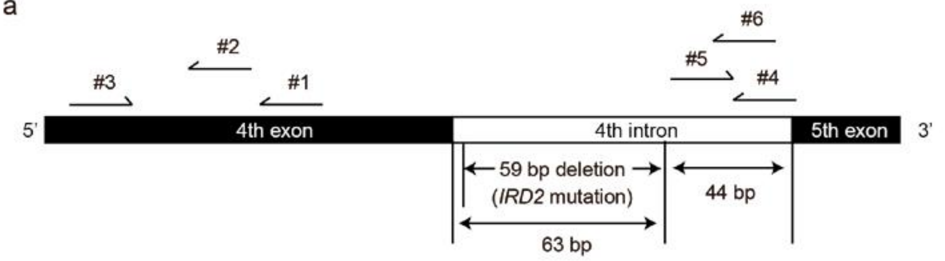

b

	sequence (21bp+PAM)	locus
#1	GGTCAAAGCAAGCTTGGATACCCGAGT	chr9:107677165-107677192
#2	ACCCGAGTCCTTCCACAAGCGCTGAAT	chr9:107677184-107677211
#3	TTGAGGAAGGCACAATGCCCAAGGAG	chr9:107677131-107677257
#4	AGCTGAAGAAGACCACCCGGTCCGAGT	chr9:107677019-107677046
#5	CACGCCACTCACCCACTCGGACCGGGT	chr9:107677033-107677060
#6	GAAGAAGACCACCCGGTCCGAGTGGGT	chr9:107677023-107677050

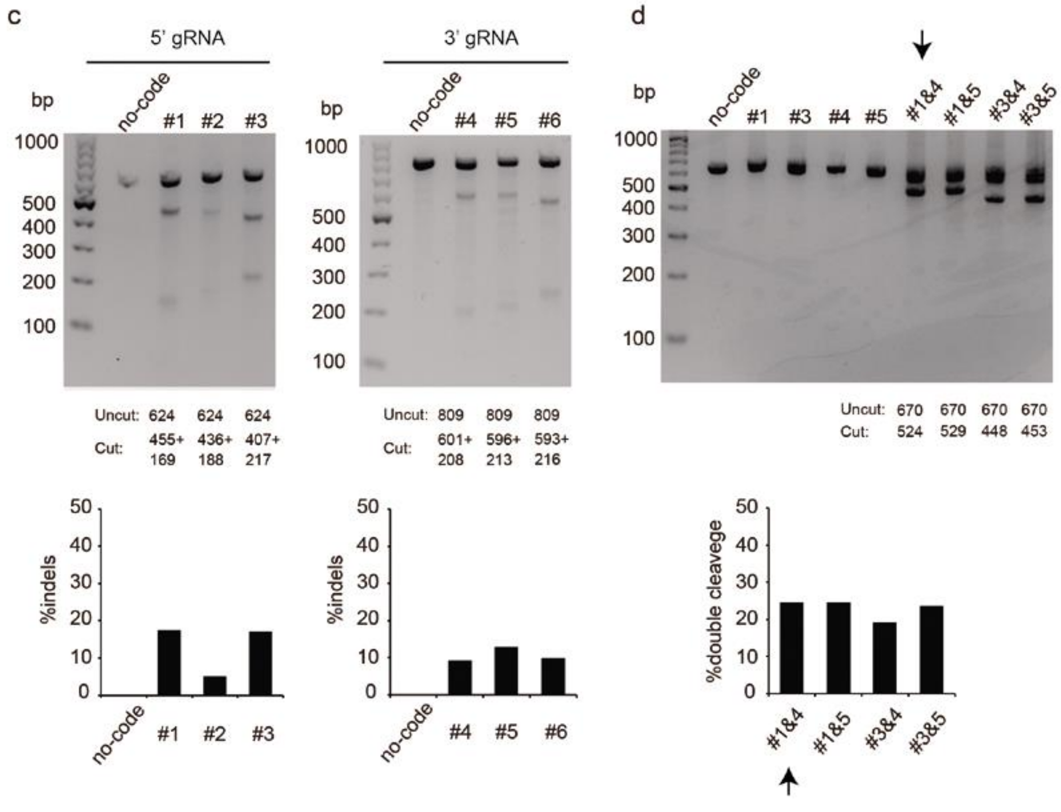

Supplementary Fig. 2| Selection of gRNA pair

a. A schematic map of gRNA designed.

b. List of gRNAs and their sequences.

c. T7E1 assay for each gRNA. The expected DNA size is displayed under the representative gel images from 3 independent replicates, all showing similar results. Quantified editing efficiency is displayed in the lower panels.

d. Electrophoresis of DNA fragments after cleavage of the genome with pairs of gRNAs. Representative images from 3 independent replicates, all showing similar results. Quantified double cleavage efficiency is displayed in the lower panel.

Source data are provided as a Source Data file.

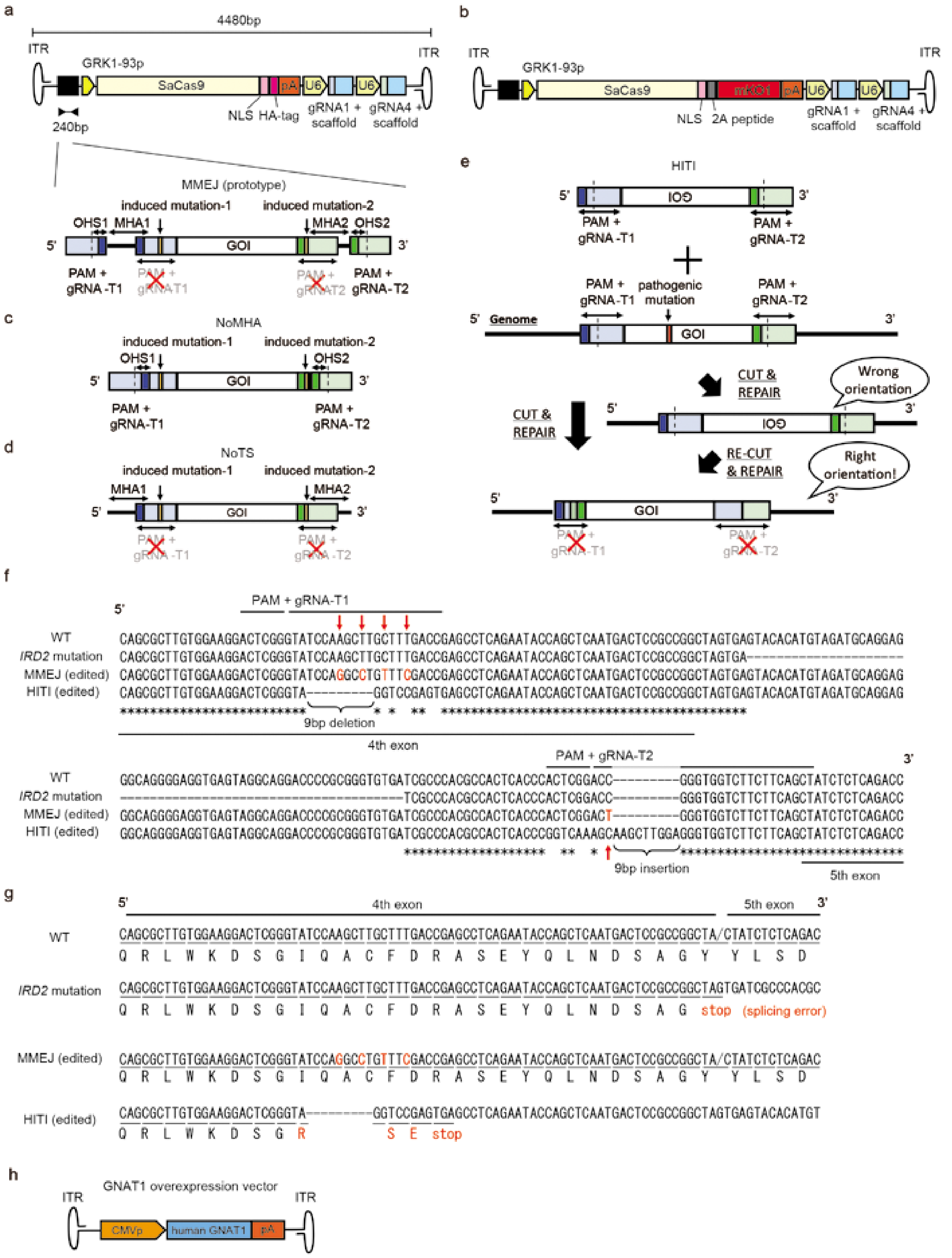

Supplementary Fig. 3| Design of genome editing vectors

a. Design the prototype MMEJ mutation replacement vector (MMEJ vector) with an enlarged

map of the donor template. Total size was 4,480 bp.

b. An enlarged map of the MMEJ vector with a reporter for lineage tracing experiments. SaCas9 and mKO1 were linked with 2A peptide. Total size was 5,201 bp.

c. An enlarged map of the donor template without flanking microhomology arms (NoMHA).

d. An enlarged map of the donor template without flanking gRNA target sites (NoTS).

e. An enlarged map of the donor template for HITI mutation replacement vector and an illustration of HITI-mediated mutation replacement strategy. In this approach, GOI was inserted in the opposite direction relative to the flanking gRNA target sites in the donor template, so that when the GOI was inserted into the genome in a correct orientation through NHEJ, these sites would be disrupted, preventing re-cleavage by SaCas9. Conversely, when the GOI were inserted in the genome in a wrong orientation, the flanking gRNA target sites would remain intact, which will be subjected to re-cleavage by SaCas9 until GOI is positioned in the correct orientation.

f. Comparison of editing outcomes at the genome level after successful applications of MMEJ- and HITI-mediated mutation replacement. Induced mutation in the gRNA target sites were highlighted in red and with arrows and nucleotides conserved across 4 sequences displayed was marked with * at the bottom of the sequence alignment. Note, 9bp deletion and 9bp insertion took place at both ends of GOI in HITI.

g. Comparison of genome editing outcomes at amino acid level after successful application of MMEJ- and HITI-mediated mutation replacement. Note, altered amino acids in reference to the wildtype sequence were highlighted in red. As a result of the significant nucleotide alterations of the 5' gRNA target site after HITI-mediated mutation replacement, 3 missense changes and 9 bp deletion followed by a nonsense mutation took place in the 4th exon.

MMEJ, micro-homology-mediated end-joining; NoMHA, no microhomology arms, NoTS, no target sites; HITI, homology-independent targeted integration; GOI, gene of interest; gRNA-T, guide RNA target; PAM, protospacer adjacent motif; ITR, inverted terminal repeat; MHA, micro homology arm; NLS, nuclear localizing signal; pA, ploy A; PAM, protospacer adjacent motif; mKO1, monomeric Kusabira-Orange 1; WT, wild-type.

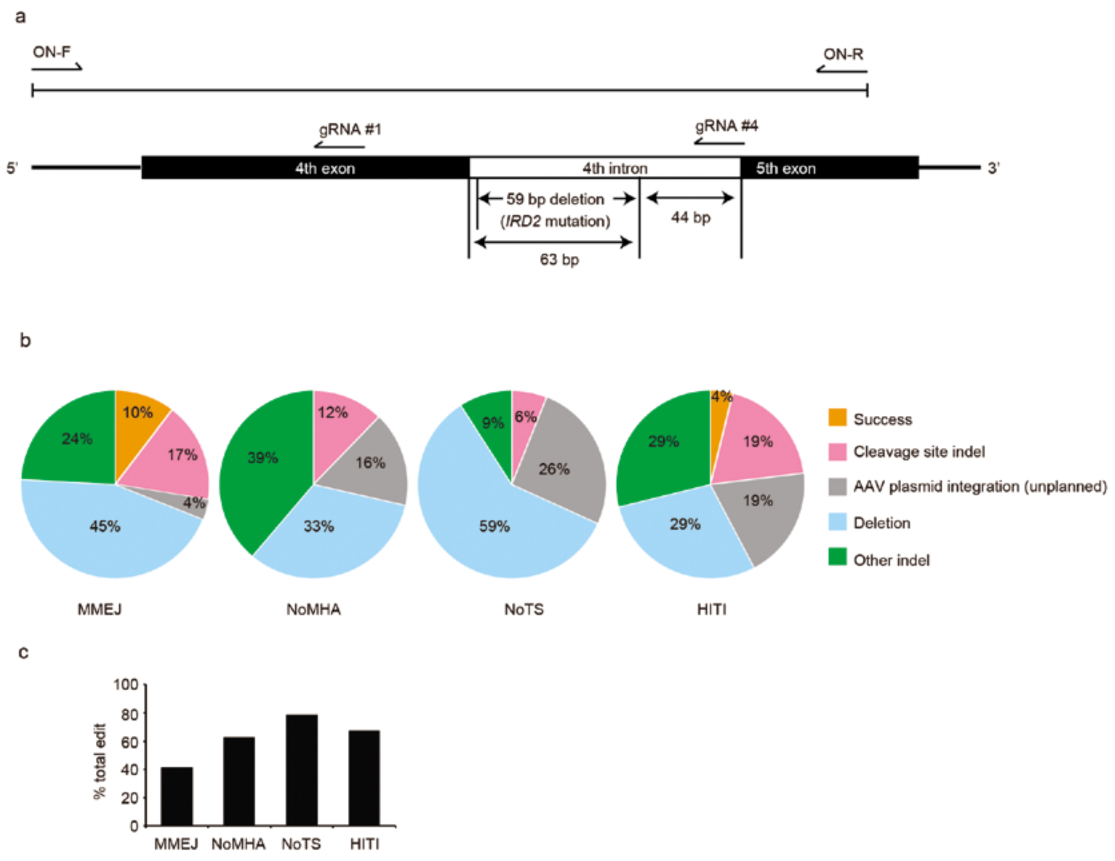

Supplementary Fig. 4| ***In vitro assessment of on-target site following mutation replacement therapy in cultured murine neural cells***

a. Schematic map of primers used for ON-target site analysis. ON-F and ON-R indicates the position of forward and reverse primer designed on the mouse genome.

b. Breakup of on-target sequencing results of the genome edited clones amplified from murine Neuro2A cell lines after transfection of the mutation replacement vector. Total clones sequenced in this experiment were 70, 67, 84 and 77 for MMEJ, NoMHA, NoTS, and HITI, respectively. The design of each vector is outlined in Supplementary Fig. 3. Note,

success indicates successful mutation replacement without induction of unplanned mutations elsewhere.

c. The rate of genome edited clones among sequenced clones.

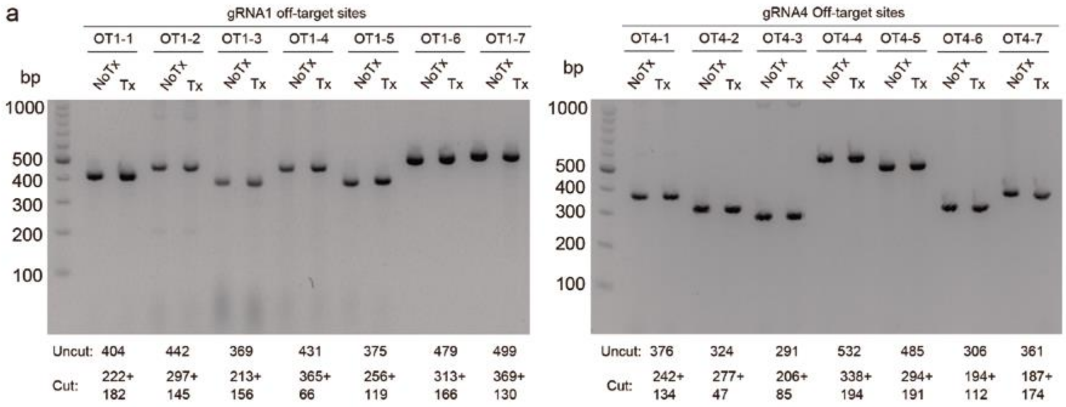

b

	site	locus	gene	sequence	CFD score	off-target clone	
						1M	3M
gRNA1	OT1-1	chr6:88651831-88651857	intergenic:Kbtbd12-Gm26588	AGTCAAAGCATGCCTGGATACTTGAGG	0.07	0/52	0/51
	OT1-2	chr8:10720942-10720968	intergenic:Myo16-Rps16-ps3	GGTCTCAGCAAGTATGGATACCTGGGT	0	0/55	0/56
	OT1-3	chr6:102298705-102298731	intron:Cntn3	GCTCAAAGAAAGATGGGATACTGGGGT	0	0/52	0/50
	OT1-4	chr18:74,959,573-74,959,599	intron:Lipg	ACCCTGATTTCCCAGCTTGCTCTGACC	0	0/55	0/51
	OT1-5	chr1:63,774,463-63,774,489	intergenic:4933402D24Rik-Gm13751	ACTCAAGTATCCCATCTGGCTTTAACC	0	0/52	0/51
	OT1-6	chr5:17999748-17999774	exon:Gnat3	TTTCAAAGCAGGCTTGGATTCCTGGAT	0	0/51	0/51
	OT1-7	chr6:83809395-83809421	intron:Paip2b	CCTCAAAGCCAGCTTGGATACCTGAAC	0	0/53	0/52
gRNA4	OT4-1	chr7:57391651-57391677	intron:Gm9962	CCTCCAACCGGGTGTGTGCCTGGTCTT	0.44	0/50	0/60
	OT4-2	chr8:126819024-126819050	intergenic:Irf2bp2-Tomm20	AGCTAAAGAAGACCACCAAGATAGGGT	0	0/50	0/54
	OT4-3	chr12:106949116-106949142	intergenic:Gm9599-Gm16087	AGCTAAAGAAGACCAGGAGGTAAGAGT	0	0/53	0/57
	OT4-4	chrX:74,257,301-74,257,327	exon:Emd	AGCTGAAGAAGGCAACCCCTTCTGGAT	0	0/56	0/50
	OT4-5	chr16:96,364,292-96,364,318	intron:Igsf5	ATTCCCTCCAGGTGGTCCTCTTCAGGT	0	0/53	0/51
	OT4-6	chr6:128843502-128843528	intergenic:Klrb1b-Gm26656	GGCTGAAGAAGGCGATCCGGTTGGGGT	0	0/50	0/50
	OT4-7	chr5:150237315-150237341	intergenic:Rxfp2-Fry	ATCTGAAGAAGCTCACCCAGTGAGAAT	0	0/50	0/52

Supplementary Fig. 5| Off-target analysis

a. T7E1 assay of 7 sites for each gRNA (total of 14 sites) predicted with CRISPOR (http://crispor.tefor.net/). Expected DNA size before (Uncut) and after (Cut) T7E1 digestion

is displayed under representative gel images from 4 independent replicates. Note that there were no bands of the expected sizes in the presence of off-target mutations.

b. Summary of off-target sites and results of Sanger sequencing. CFD scores represent the likelihood of off-target DNA damage induction. The numbers of sequenced clones and mutations found (all zero) are expressed as denominators and numerators, respectively, in the column off-target clone. OT, off-target. Tx, treated with T7E1; NoTx, Not treated. Source data are provided as a Source Data file.

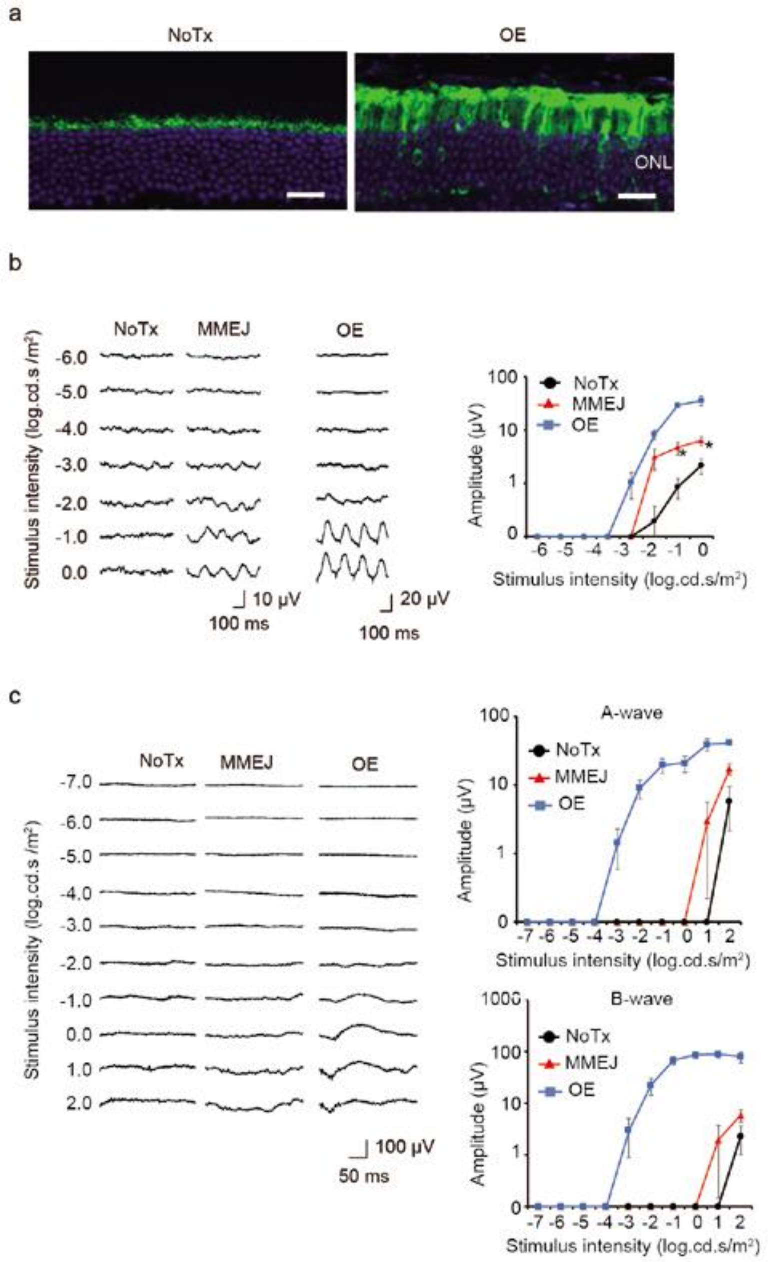

Supplementary Fig. 6| Comparison of MMEJ-mediated mutation replacement and gene supplementation therapy in *Pde6c*$^{cpfl1/cpfl1}$*Gnat1*$^{IRD2/IRD2}$ mice

a. Representative GNAT1 immunohistochemistry images of the retina in *Pde6c*$^{cpfl1/cpfl1}$*Gnat1*$^{IRD2/IRD2}$ mice treated and untreated with *GNAT1* over-expression.

b. 6Hz flicker ERGs recorded from the eyes treated with either MMEJ-mediated *Gnat1*-

IRD2 mutation replacement (MMEJ, N = 9) or *GNAT1* over-expression (OE, N = 9) and the untreated (NoTx, N = 6) eyes of *Pde6c*$^{cpfl1/cpfl1}$*Gnat1*$^{IRD2/IRD2}$ mice.

c. Single flash ERGs recorded from the eyes treated with either MMEJ-mediated *Gnat1-IRD2* mutation replacement (MMEJ, N = 7) or *GNAT1* over-expression (OE, N = 7) and the untreated (NoTx, N = 6) eyes of *Pde6c*$^{cpfl1/cpfl1}$*Gnat1*$^{IRD2/IRD2}$ mice.

Data represent the mean ± S.E.M.; *P < 0.05 (Student's t-test) for comparison between NoTX and MMEJ. MMEJ, micro-homology-mediated end-joining. Source data are provided as a Source Data file.

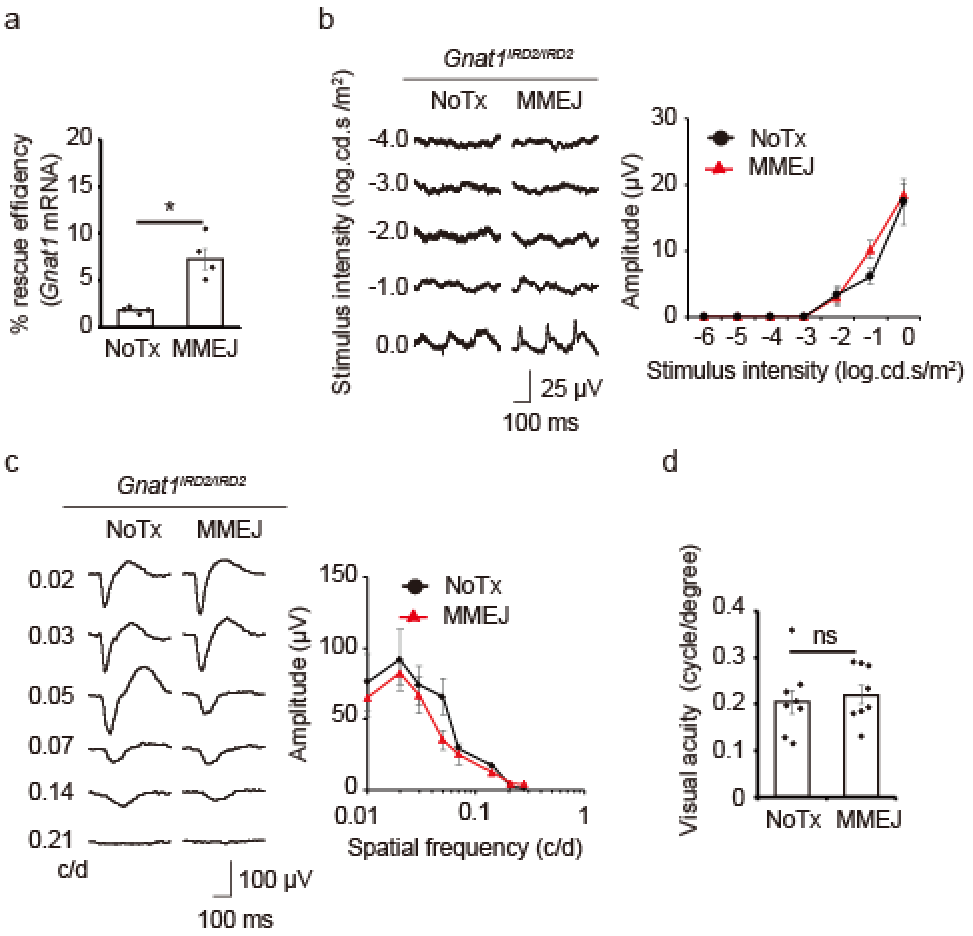

Supplementary Fig. 7| Treatment of *Gnat1$^{IRD2/IRD2}$* mice with MMEJ-mediated mutation replacement AAV vector

a, Genome editing efficiency as measured with RT-PCR (N = 5) in *Gnat1$^{IRD2/IRD2}$* mice. **b, c, d**. Flicker ERGs (**b**, N = 7), OKR (**c**, N = 8) and pVEPs (**d**, N = 7) detected no treatment effect.

Data represent the mean ± S.E.M.; *$P < 0.05$ (Student's t-test); MMEJ, micro-homology-mediated end-joining; NoTx untreated; ns, not significant. Source data are provided as a Source Data file.

Supplementary Table 1. Promoter sequences

Promoter	Sequence (5’>3’)
b-actin	GTCGAGGTGAGCCCCACGTTCTGCTTCACTCTCCCCATCTCCCCCCCCTCCCCACCCCCAATTT<br>TGTATTTATTTATTTTTTAATTATTTTATGCAGCGATGGGGGCGGGGGGGGGGGGGGCGCGCGC<br>CAGGCGGGGCGGGGCGGGGCGAGGGGCGGGGCGGGGCGAGGCGGAGAGGTGCGGCGGCA<br>GCCAATCAGAGCGGCGCGCTCCGAAAGTTTCCTTTTATGGCGAGGCGGCGGCGGCGGCGGCC<br>CTATAAAAAGCGAAGCGCGCGGCGGGCGGGAGTCGCTGCGTTGCCTTCGCCCCGTGCCCCGC<br>TCCGCGCCGCCTCGCGCCGCCCGCCCCGGCTCTGACTGACCGCGTTACTCCCACAG
CMV mini	GGTAGGCGTGTACGGTGGGAGGCCTATATAAGCAGAGCTCGTTTAGTGAACCGTCAGATCGCCT<br>GGAG
CMV-s	CGTTACATAACTTACGGTAAATGGCCCGCCTGGCTGACCGCCCAACGACCCCCGCCCATTGACG<br>TCAATAATGACGTATGTTCCCATAGTAACGCCAATAGGGACTTTCCATTGACGTCAATGGGTGGA<br>GTATTTACGGTAAACTGCCCACTTGGCAGTACATCAAGTGTATCATATGCCAAGTACGCCCCCTA<br>TTGACGTCAATGACGGTAAATGGCCCGCCTGGCATTATGCCCAGTACATGACCTTATGGGACTTT<br>CCTACTTGGCAGTACATCTACGTATTAGTCATCGCTATTACCatgAGAGGGTATATAATGGAAGCT<br>CGACTTCCAG
EFS	TGGCTCCGGTGCCCGTCAGTGGGCAGAGCGCACATCGCCCACAGTCCCCGAGAAGTTGGGGG<br>GAGGGGTCGGCAATTGAACCGGTGCCTAGAGAAGGTGGCGCGGGGTAAACTGGGAAAGTGAT<br>GTCGTGTACTGGCTCCGCCTTTTTCCCGAGGGTGGGGGAGAACCGTATATAAGTGCAGTAGTCG<br>CCGTGAACGTTCTTTTTCGCAACGGGTTTGCCGCCAGAACACAGGT
GRK-199	GGGCCCCAGAAGCCTGGTGGTTGTTTGTCCTTCTCAGGGGAAAAGTGAGGCGGCCCCTTGGAG<br>GAAGGGGCCGGGCAGAATGATCTAATCGGATTCCAAGCAGCTCAGGGGATTGTCTTTTTCTAGC<br>ACCTTCTTGCCACTCCTAAGCGTCCTCCGTGACCCCGGCTGGGATTTAGCCTGGTGCTGTGTCA<br>GCCCCGGG
GRK-174	TGTCCTTCTCAGGGGAAAAGTGAGGCGGCCCCTTGGAGGAAGGGGCCGGGCAGAATGATCTAA<br>TCGGATTCCAAGCAGCTCAGGGGATTGTCTTTTTCTAGCACCTTCTTGCCACTCCTAAGCGTCCT<br>CCGTGACCCCGGCTGGGATTTAGCCTGGTGCTGTGTCAGCCCCGGG
GRK-94	TCTCAGGGGATCTAATCGGATTAGCAGCTAGGGGATTGTCTTTTTCTGCACCTTCTCCTAAGGTC<br>CTCCGTGACCCCGGATTTAGTGTCAGCCC
GRK-93	TCTAATCGGATTCCAAGCAGCTCAGGGGATTGTCTTTTTCTAGCACCTTCTTGCCACTCCTAAGC<br>GTCCTCCGTGACCCCGGCTGGGATTTAG
Mecp2	AGCTGAATGGGGTCCGCCTCTTTTCCCTGCCTAAACAGACAGGAACTCCTGCCAATTGAGGGCG<br>TCACCGCTAAGGCTCCGCCCCAGCCTGGGCTCCACAACCAATGAAGGGTAATCTCGACAAAGA<br>GCAAGGGGTGGGGCGCGGGCGCGCAGGTGCAGCAGCACACAGGCTGGTCGGGAGGGCGGG<br>GCGCGACGTCTGCCGTGCGGGGTCCCGGCATCGGTTGCGCGC
Syn1	CTGTGAGGGGGTTATTTCTCTACTTTCGTGTCTCTGAGTGTGCTTCCAGTGCCCCCCTCCCCCCA<br>AAAAATGCCTTCTGAGTTGAATATCAACACTACAAACCGAGTATCTGCAGAGGGCCCTGCGTATG<br>AGTGCAAGTGGGTTTTAGGACCAGGATGAGGCGGGGTGGGGGTGCCTACCTGACGACCGACC<br>CCGACCCACTGGACAAGCACCCAACCCCCATTCCCCAAATTGCGCATCCCCTATCAGAGAGGG<br>GGAGGGGAAACAGGATGCGGCGAGGCGCGTGCGCACTGCCAGCTTCAGCACCGCGGACAGTG<br>CCTTCGCCCCCGCCTGGCGGCGCGCGCCACCGCCGCCTCAGCACTGAAGGCGCGCTGACGTC<br>ACTCGCCGGTCCCCCGCAAACTCCCCTTCCCGGCCACCTTGGTCGCGTCCGCGCCGCCGCCG<br>GCCCAGCCGGACCGCACCACGCGAGGCGCGAGATAGGGGGGCACGGGCGCGACCATCTGCG<br>CTGCGGCGCCGGCGACTCAGCGCTGCCTCAGTCTGCGGTGGGCAGCGGAGGAGTCGTGTCGT<br>GCCTGAGAGCGCAG
RCV-845	TCAGACATATTGACTCACATCAGCCTCACAATGACAGTGTGGTAGATGCTATGATGCCCATTTATT<br>CAAGAAAGACTTGCTGGGGGCCCAAGCCTATCAGGTTCTGGCAATGGAAATGCATCCGGGAACA<br>ACACAGACAAAATCCCATCCTTCACGGAGCTTTCATTCCGGTGAGGGGACATGCAGCGTGCCGA<br>TGATGGTGGGGGTTGTGCTATTATAGATAGAGGGTCAGTATAGGCCTGGCTGAGCAGGTGACAT<br>TTAAGCAGAGACATGACTGCAGTGAAGGTTTAGAAGAGTGTCCCAGGTGGAAAGAGGTGAAGAA<br>ACAAGCCTGGGGCCACACAGCTAGTTAAGTAGCCAAGCTGGGACTTGAACCCATGCTGGCCCC<br>AGAGTCTGTGCTCCTAACCATTGCATTCTAGGGCTTGATATGAGATGCCAGCCCCGCCCCGAGA<br>TGCTCAGAGTTAGTGAGGAAGAGAAACAGGGAACATGGCTGCTGTTAGAGGGCTGGGGCTGGG<br>GGTGCCGGAGGCCCCAGCTCTGAGGGTTCCAACCTCCTGTCCTGTTCTAGTATCGTCCCGGGA<br>GGCCGAGATGAATTGCCTGCCTGCCCTGGGCTCTTTATTTTAATCTCACTAGGGTTCTGGGAGC<br>ACCCCCCCCCACCGCTCCCGCCCTCCACAAAGCTCCTGGGCCCCTCCTCCCTTCAAGGATTGC<br>GAAGAACTGGTCGCAAATCCTCCTAAGCCACCAGCATCTCGGTCTTCAGCTCACACCAGCCTTG<br>AGCCCAGCCTGCGGCCAGGGGACCACGCACGTCCCACCCACCCAGCGACTCCCCAGCCGCTG<br>CCCACTCTTCCTCACTC

RCV-223	ACTAGGGTTCTGGGAGCACCCCCCCCCACCGCTCCCGCCCTCCACAAAGCTCCTGGGCCCCTC CTCCCTTCAAGGATTGCGAAGAACTGGTCGCAAATCCTCCTAAGCCACCAGCATCTCGGTCTTC AGCTCACACCAGCCTTGAGCCCAGCCTGCGGCCAGGGGACCACGCACGTCCCACCCACCCAG CGACTCCCCAGCCGCTGCCCACTCTTCCTCACTC
RCV-111	ATATTGACTCACATGGCAATGGAAATGCTCTGAGGGTTCCAACCTCCTGTCCTGTTCTGCCTGCC CTGGGCTCTTTATTTTAATCTCACTAGGGCCCCTCCTCCCTTCAAG
RCV-92	ATATTGACTCACAGCTCTGAGGGTTCCAACCTCCTGTCCTGTTCTGCCTGCCCTGGGCTCTTTAT TTTAATCTCACCCCTCCTCCCTTCAAG

Supplementary Table 2. Primer sequences for on- and off-target analysis and RT-PCR

Primer ID	Sequence (5’>3’)	Use
ON-F (Fig. 2, Fig. S3c,d,Fig. S4)	CAGATGAAGTGAGTGTTCCCTG	On-target
ON-R1 (Fig. S3c right panel)	ACCTTCACTCAAGACACTGACG	On-target
ON-R2 (Fig. S3c left panel)	GAGATAGCTGAAGAAGACCACCC	On-target
ON-R3(Fig. 2, Fig. S3d, Fig. S4)	GCTCAGTGGGCACATATCCTG	On-target
OT1-1F	TGCATCTGCCACCTCTGAAG	Off-target
OT1-1R	TCACAGTCACAGAACCAGGC	Off-target
OT1-2F	TCTCAAGGAGCCCAGAGTGA	Off-target
OT1-2R	ACTTACTCGAGGGGCAGCTA	Off-target
OT1-3F	ATGCTGAGAGCTGATGCTCC	Off-target
OT1-3R	GGTTAACCACAGGGCTCAGG	Off-target
OT1-4F	TGAGACACCATCCCCTACCC	Off-target
OT1-4R	CCCAGAAGCCAAAGTGACCT	Off-target
OT1-5F	CTCATGGGTCTTTTGCCTTTCC	Off-target
OT1-5R	ATTCATGTATGGGAAGGCAGTG	Off-target
OT1-6F	GTGCCTGTGCCTAAATAGCAT	Off-target
OT1-6R	CCAAATATCAACACGCATGCCA	Off-target
OT1-7F	AGGCTGTTCCTTCATGTGTCC	Off-target
OT1-7R	CCTTAGATGAGCCCCAAGCTC	Off-target
OT4-1F	ACACAGCTAAGCATCAGGCAGAG	Off-target
OT4-1R	TGTGTCTACCACAAGTGCCC	Off-target
OT4-2F	GGGTTTTGAAATACTAACCACATGG	Off-target
OT4-2R	AGGTAAGTCTCTTGGGGGAAG	Off-target
OT4-3F	TGGTAGCAGATAAGGAAGGGT	Off-target
OT4-3R	CAGATGTCCCACAGCTGCTT	Off-target
OT4-4F	CTACCTGTTGCCTTCTTTTGCC	Off-target
OT4-4R	GGCCAACATTTGCTGCTTCC	Off-target
OT4-5F	TTCAGAAGCAAGGCAGGATGA	Off-target
OT4-5R	TCCAGACAGAGGAAACGCCT	Off-target
OT4-6F	ATCCACGGTTATACTGCGCC	Off-target
OT4-6R	CATTGTGGTGCAGGGCTAGA	Off-target
OT4-7F	CAAGTCCCAGCCTTCCTGAG	Off-target
OT4-7R	ACACCAGCTTAACTGCCTGA	Off-target
SaCas9F	GTGGCACACCAACGACAAC	qRT-PCR
SaCas9R	TCTTGGGCACCAGCTTCAG	qRT-PCR
SaCas9probe	FAM-CCGGTTGAAGATAGCG-NFQ	qRT-PCR

Supplementary Table 3. List of off-target sites analyzed with whole genome sequencing

Category	Region	GeneSymbol	Chr	Coordinate	Read depth NoTx-1M (N=1)	Read depth MMEJ-1M (N=4)	Read depth MMEJ-3M (N=3)
OT1-1	intergenic	-	chr6	88651831-88651857	44	157	124
OT1-2	intergenic	-	chr8	10720942-10720968	42	155	103
OT1-3	intronic	Cntn3	chr6	102298705-102298731	55	144	114
OT1-4	intronic	Lipg	chr18	74959573-74959599	47	175	129
OT1-5	intergenic	-	chr1	63774463-63774489	38	161	142
OT1-6	exonic	Gnat3	chr5	17999748-17999774	35	151	124
OT1-7	intronic	Paip2b	chr6	83809395-83809421	49	160	122
OT4-1	intronic	Gm9962	chr7	57391651-57391677	43	139	134
OT4-2	intergenic	-	chr8	126819024-126819050	57	157	118
OT4-3	intergenic	-	chr12	106949116-106949142	32	191	164
OT4-4	exonic	Emd	chrX	74257301-74257327	33	150	129
OT4-5	intronic	Igsf5	chr16	96364292-96364318	45	148	91
OT4-6	intergenic	-	chr6	128843502-128843528	55	162	144
OT4-7	intergenic	-	chr5	150237315-150237341	47	168	125
unbiased	intergenic	-	chr1	164605179	34	131	89
unbiased	intergenic	-	chr2	7868516	25	85	88
unbiased	intergenic	-	chr2	15297474	25	125	87
unbiased	intronic	Etl4	chr2	20713166	27	109	98
unbiased	intergenic	-	chr2	43051827	31	114	108
unbiased	intergenic	-	chr2	80770531	39	123	102
unbiased	intergenic	-	chr2	97914246	31	113	104
unbiased	intergenic	-	chr2	99501578	39	109	108
unbiased	intergenic	-	chr3	72739035	35	118	104
unbiased	intronic	Bche	chr3	73697230	38	117	105
unbiased	intronic	Trabd2b	chr4	114414263	36	115	101
unbiased	intergenic	-	chr4	144174561	11	41	27
unbiased	intergenic	-	chr4	144174568	10	40	28
unbiased	intronic	Cacna2d1	chr5	15960665	41	127	88
unbiased	intergenic	-	chr5	56595988	38	122	129
unbiased	intergenic	-	chr5	97210056	48	124	120
unbiased	intergenic	-	chr6	104422359	38	119	106
unbiased	intergenic	-	chr7	23538806	36	132	104
unbiased	intronic	Luzp2	chr7	54892475	38	115	77
unbiased	intergenic	-	chr7	65527497	43	127	128
unbiased	intronic	Xpo6	chr7	126148059	44	133	111
unbiased	intergenic	-	chr8	7685578	9	36	21
unbiased	intergenic	-	chr8	7685580	9	36	21
unbiased	intergenic	-	chr8	30227306	31	136	97
unbiased	intergenic	-	chr8	37274525	26	141	110
unbiased	intergenic	-	chr9	17682682	34	127	97
unbiased	intergenic	-	chr9	18880190	36	121	106
unbiased	intergenic	-	chr9	24707398	38	116	111
unbiased	intronic	Ice2	chr9	69422497	42	131	98
unbiased	intergenic	-	chr9	110410881	26	71	49
unbiased	intergenic	-	chr9	117753855	30	114	102
unbiased	intergenic	-	chr10	11935392	41	136	110
unbiased	intergenic	-	chr10	12107120	37	130	99
unbiased	intergenic	-	chr10	15154603	37	99	96
unbiased	intergenic	-	chr10	16537474	33	141	112
unbiased	intergenic	-	chr10	88194335	31	136	119
unbiased	intergenic	-	chr12	113592895	33	84	104
unbiased	intergenic	-	chr12	113592901	33	83	104
unbiased	intergenic	-	chr13	4392659	37	170	121
unbiased	intronic	Adarb2	chr13	8318995	20	119	98
unbiased	intronic	Aopep	chr13	63065021	34	126	104
unbiased	intergenic	-	chr13	71861889	10	130	70
unbiased	intergenic	-	chr13	75061692	34	136	101
unbiased	intronic	Serf1	chr13	100111981	44	133	117
unbiased	intergenic	-	chr14	70988337	30	95	73
unbiased	intronic	Vps13b	chr15	35907409	34	136	93
unbiased	intergenic	-	chr16	3237085	86	359	263

unbiased	intronic	Srl	chr16	4505718	32	94	104
unbiased	intronic	Cblb	chr16	52032376	26	112	93
unbiased	intergenic	-	chr16	63070898	34	98	97
unbiased	intergenic	-	chr16	72053890	33	131	125
unbiased	intergenic	-	chr16	72419608	36	122	110
unbiased	intronic	Vps52	chr17	33961985	23	130	94
unbiased	intergenic	-	chr17	34497571	28	141	94
unbiased	intergenic	-	chr17	36300692	35	123	106
unbiased	intronic	Myom1	chr17	71080552	35	130	109
unbiased	intronic	Gm37013,Gm38666, Gm38667	chr18	37364415	37	175	148
unbiased	intergenic	-	chr18	42391008	35	86	81
unbiased	intronic	Lipn	chr19	34075362	31	134	90
