# Supplementary material for: Single AAV-mediated mutation replacement genome editing in limited number of photoreceptors restores vision in mice
Source: Nat Commun. 2020 Jan 24;11:482. doi: 10.1038/s41467-019-14181-3 (PMC6981188; doi:10.1038/s41467-019-14181-3)
Supplement: Supplementary file 3 — Reporting Summary [file 41467_2019_14181_MOESM3_ESM.pdf]

nature research

Corresponding author(s): Koji M Nishiguchi

Last updated by author(s): Dec 10, 2019

Reporting Summary

Nature Research wishes to improve the reproducibility of the work that we publish. This form provides structure for consistency and transparency in reporting. For further information on Nature Research policies, see Authors & Referees and the Editorial Policy Checklist .

Statistics

For all statistical analyses, confirm that the following items are present in the figure legend, table legend, main text, or Methods section.

n/a	Confirmed	
☐	☒	The exact sample size (*n*) for each experimental group/condition, given as a discrete number and unit of measurement
☐	☒	A statement on whether measurements were taken from distinct samples or whether the same sample was measured repeatedly
☐	☒	The statistical test(s) used AND whether they are one- or two-sided<br>*Only common tests should be described solely by name; describe more complex techniques in the Methods section.*
☒	☐	A description of all covariates tested
☒	☐	A description of any assumptions or corrections, such as tests of normality and adjustment for multiple comparisons
☐	☒	A full description of the statistical parameters including central tendency (e.g. means) or other basic estimates (e.g. regression coefficient) AND variation (e.g. standard deviation) or associated estimates of uncertainty (e.g. confidence intervals)
☒	☐	For null hypothesis testing, the test statistic (e.g. F, t, r) with confidence intervals, effect sizes, degrees of freedom and P value noted<br>*Give P values as exact values whenever suitable.*
☒	☐	For Bayesian analysis, information on the choice of priors and Markov chain Monte Carlo settings
☒	☐	For hierarchical and complex designs, identification of the appropriate level for tests and full reporting of outcomes
☒	☐	Estimates of effect sizes (e.g. Cohen's *d*, Pearson's *r*), indicating how they were calculated

Our web collection on statistics for biologists contains articles on many of the points above.

Software and code

Policy information about availability of computer code

Data collection	Off-target sites were predicted by CRISPOR (http://crispor.tefor.net/) .<br>gRNAs were designed using the Cas-Designer (http://www.rgenome.net/cas-designer/).<br>gRNA target site mutations were designed using theGENEisu (http://www.geneius.de/GENEius/).<br>Sequences were aligned by ClustalW (https://clustalw.ddbj.nig.ac.jp/)<br>A sample size calculator was used (https://www.stat.ubc.ca/).<br>Gel bands intensity were measured by Image J 1.51 (https://imagej.nih.gov/ij/).
Data analysis	Statistical analysis was performed by JMP Pro 13.10 (SAS Institute, Cary, NC).

For manuscripts utilizing custom algorithms or software that are central to the research but not yet described in published literature, software must be made available to editors/reviewers. We strongly encourage code deposition in a community repository (e.g. GitHub). See the Nature Research guidelines for submitting code & software for further information.

Data

Policy information about availability of data

All manuscripts must include a data availability statement. This statement should provide the following information, where applicable:

- Accession codes, unique identifiers, or web links for publicly available datasets
- A list of figures that have associated raw data
- A description of any restrictions on data availability

The source data underlying Figs 1d-h, 2b–h, 3a-d, 4b and c and Supplementary Figs 1d,f, 2c, 5a, 6b, c and 7a-d are provided as a Source Data file. The datasets generated during and/or analyzed during the current study not listed in the Source Data file are available from the corresponding author on reasonable request.

Field-specific reporting

Please select the one below that is the best fit for your research. If you are not sure, read the appropriate sections before making your selection.

- [x] Life sciences
- [ ] Behavioural & social sciences
- [ ] Ecological, evolutionary & environmental sciences

For a reference copy of the document with all sections, see nature.com/documents/nr-reporting-summary-flat.pdf

Life sciences study design

All studies must disclose on these points even when the disclosure is negative.

Sample size	Sample sizes were calculated using on-line sample size calculator (https://www.stat.ubc.ca/) adopting a two-sided alpha-level of 0.05, 80% power, with the parameters including the means and common standard deviation predicted from our previous study (ref 9) using the same group of mice and similar experimental approach. Rarely, experiment size was restricted by the availability of the mice.
Data exclusions	Occasional mice with corneal opacity in either of the eyes were excluded from the study prior to the treatment assignment.
Replication	For each series of experiments, all replication attempts were successful.
Randomization	All animals were randomly assigned to treatment or non-treatment.
Blinding	The researcher responsible for intraocular injection was not blinded to the status, i.e., treatment vs non-treatment, as no sham treatment was carried out. However, although the following electrophysiological assessments were objective tests, the assignment of the treatment status became unintentionally obvious due to the clear-cut treatment effects detected. Therefore, the behavioral experiments were carried out by a technichian blinded to the results of electrophysiological tests with minimal knowledge of the experimental design.

Reporting for specific materials, systems and methods

We require information from authors about some types of materials, experimental systems and methods used in many studies. Here, indicate whether each material, system or method listed is relevant to your study. If you are not sure if a list item applies to your research, read the appropriate section before selecting a response.

Materials & experimental systems

n/a	Involved in the study	
[ ]	[x]	Antibodies
[ ]	[x]	Eukaryotic cell lines
[x]	[ ]	Palaeontology
[ ]	[x]	Animals and other organisms
[x]	[ ]	Human research participants
[x]	[ ]	Clinical data

Methods

n/a	Involved in the study	
[x]	[ ]	ChIP-seq
[x]	[ ]	Flow cytometry
[x]	[ ]	MRI-based neuroimaging

Antibodies

Antibodies used	anti-GNAT1 (ab74059, 1/2,000; Abcam, Cambridge, UK)<br>anti-GFP antibody (#598, 1/2500; MBL, Nagoya, JAPAN)<br>anti-mKO1 antibody (#M104-3M, 1/200; MBL, Nagoya, JAPAN)<br>anti-beta-actin (F5316, 1/2000; Sigma-Aldrich, St. Louis, MO)<br>HRP-conjugated anti-rabbit IgG antibodies (A0545, 1/2000; Sigma-Aldrich)<br>HRP-conjugated anti-mouse antibodies (#31430, 1/2000; Thermo Fisher Scientific, Waltham, MA)<br>Alexa Fluo 568-conjugated anti-mouse IgG antibodies (#A-11004, 1/500; Thermo Fisher Scientific)
Validation	The antibodies have been validated by their manufacturers as described on the company website.<br>The specificity of the antibody has been confirmed by western blot showing a band corresponding to the expected size following overexpression of the GNAT1 cDNA in our previous study (ref 9).

Eukaryotic cell lines

Policy information about cell lines

Cell line source(s)	Neuro2A: Cell Resource Center for Biomedical Research, Tohoku University, Sendai, Japan<br>HEK293T: Thermo Fisher Scientific, Waltham, MA

Authentication	The origin of the HEK293T cells was identified as human by PCR.<br>The origin of the Neuro2A was identified as mouse by whole genome sequencing.<br>HEK293T was used to produce AAV but not for other purpose
Mycoplasma contamination	The cells used were mycoplasma free. We test cells routinely for mycoplasma contamination.
Commonly misidentified lines<br>(See ICLAC register)	No commonly misidentified cell lines were used.

Animals and other organisms

Policy information about studies involving animals; ARRIVE guidelines recommended for reporting animal research

Laboratory animals	C57BL/6J mice were provided from Japan SLC Inc., Hamamatsu, Japan<br>Gnat1IRD2/IRD2 mice were provided from Takeda, Japan (ref 1, on-line Methods)<br>Pde6c cpfl1/cpfl1 mice were purchased from the Jackson Laboratory (Bar Harbor, ME) . The phenotype of the mice has been described (ref 2, on-line Methods).<br>The age of mice used for each line is designated in the main text.
Wild animals	No wild animals were used in this study.
Field-collected samples	Study did not involve specimens collected from the field.
Ethics oversight	All experimental procedures were conducted after approval by the related committees, including animal ethics committee, for the animal experiments at Tohoku University Graduate School of Medicine.

Note that full information on the approval of the study protocol must also be provided in the manuscript.
